# Mechanisms of Severe Mortality-Associated Bacterial Co-infections Following Influenza Virus Infection

**DOI:** 10.3389/fcimb.2017.00338

**Published:** 2017-08-03

**Authors:** Leili Jia, Jing Xie, Jiangyun Zhao, Dekang Cao, Yuan Liang, Xuexin Hou, Ligui Wang, Zhenjun Li

**Affiliations:** ^1^Institute of Disease Control and Prevention of Chinese People's Liberation Army Beijing, China; ^2^Center for Disease Control and Prevention of Chinese People's Armed Police Forces Beijing, China; ^3^State Key Laboratory for Infectious Disease Prevention and Control, National Institute for Communicable Disease Control and Prevention, Chinese Center for Disease Control and Prevention Beijing, China

**Keywords:** co-infection, influenza, bacteria, resumption of function, tolerance, mortality

## Abstract

Influenza virus infection remains one of the largest disease burdens on humans. Influenza-associated bacterial co-infections contribute to severe disease and mortality during pandemic and seasonal influenza episodes. The mechanisms of severe morbidity following influenza-bacteria co-infections mainly include failure of an antibacterial immune response and pathogen synergy. Moreover, failure to resume function and tolerance might be one of the main reasons for excessive mortality. In this review, recent advances in the study of mechanisms of severe disease, caused by bacterial co-infections following influenza virus pathogenesis, are summarized. Therefore, understanding the synergy between viruses and bacteria will facilitate the design of novel therapeutic approaches to prevent mortality associated with bacterial co-infections.

## Introduction

Influenza viruses are responsible for an average of 400,000 deaths per year globally (Simonsen et al., 1997; Bakaletz, 2004; King et al., 2017; Tansey, 2017). During previous influenza pandemics (H1N1, 1918; H2N2, 1957; H3N2, 1968; H1N1, 2009) and seasonal epidemics, many influenza-related deaths actually occurred due to bacterial co-infections (Guarner et al., 2006; Taubenberger and Morens, 2008; Weiser, 2010; Klein et al., 2016; McDanel et al., 2016; Shah et al., 2016). Since the 1950s, researchers have increasingly focused on concomitant infections with influenza viruses and a range of bacterial agents (Table 1).

**Table 1:** Bacterial co-infection with influenza.

**Influenza strain**	**Bacteria**	**Gram-stain**	**References**
H1N1	*Streptococcus pneumoniae (S. pneumoniae)*	Positive	Morens et al., 2008
	*Streptococcus pyogenes (S. pyogenes)* group *(A Streptococcus)*	Positive	Schwarzmann et al., 1971
	*Methicillin resistant staphylococcus aureus (MRSA)*	Positive	Hageman et al., 2006
	*Mycobacterium tuberculosis (M. tuberculosis)*	Positive	Tan et al., 2011; Park et al., 2014; Alghamdi et al., in press
	*Meningococci*	Negative	Reilly and Gaunt, 1991; Legriel et al., 2011
	*Legionella pneumophila*	Negative	Iannuzzi et al., 2011
	*Staphylococcal aureus (S. aureus)*	Positive	Takayama et al., 2014; Park et al., 2015; Gabrilovich et al., 2017
H2N2	*Streptococcus pneumoniae (S. pneumoniae)*	Positive	Oseasohn et al., 1959
	*Staphylococcal aureus (S. aureus)*	Positive	Petersdorf et al., 1959
	*Haemophilus influenzae*	Negative	Watt et al., 2009
H3N2	*Streptococcus pneumoniae (S. pneumoniae)*	Negative	Schwarzmann et al., 1971
	*Staphylococcal aureus (S. aureus)*	Positive	Kobayashi et al., 2013; Collins et al., 2017
	*Campylobacter jejuni*	Negative	Kahar-Bador et al., 2009
H7N9	*Legionella pneumophila*	Negative	Gao et al., 2013
	*Klebsiella pneumoniae*	Negative	Gao et al., 2013
	*Acinetobacter baumannii*	Negative	Gao et al., 2013
	*Burkholderia cepacia*	Negative	Gao et al., 2013
	*Pseudomonas aeruginosa*	Negative	Gao et al., 2013
	*Enterobacter aerogenes*	Negative	Gao et al., 2013
	*K. oxytoca*	Negative	Gao et al., 2013
	*P. putida*	Negative	Gao et al., 2013
	*Staphylococcal aureus (S. aureus)*	Positive	Yang et al., 2016

Mortality incidence can be affected by several factors, one of which being the order of co-infections. Although it is difficult to distinguish the order in which bacterial and influenza infection occurs in a clinical setting, laboratory data have shown that mortality is associated with this sequence. Specifically, mortality incidence peaks when bacterial infections occur 3–7 days after an established influenza infection (Jamieson et al., 2013). In this review, we mainly address the mechanisms of severe morbidity and mortality associated with bacterial co-infections following influenza infection.

## Influenza infection increases host susceptibility to bacteria

In both humans and mice, influenza virus titers in the lung reach a peak 3–5 days after primary infection is established. Thereafter, the virus clearance begins, with almost complete resolution of infection between 10 and 12 days (Metzger and Sun, 2013). In general, the influenza virus preferentially replicates in epithelial cells and induces the most lung tissue damage at approximately day 6 (Nugent and Pesanti, 1983). This pathology is partly responsible for the observed increase in susceptibility to opportunistic bacterial pathogens, as epithelial cell damage and increased receptor availability enable invading bacteria to adhere and grow.

### Influenza affects the antibacterial innate immune response

Typically, the respiratory tract immune system is strictly controlled to prevent inflammation in response to innocuous antigens or commensal bacteria. When harmful pathogens colonize the respiratory tract, the local immune system becomes activated to eliminate the threat. It was found that typical mice can effectively clear up to 10^5^ pneumococci within 4–12 h (Sun and Metzger, 2008). However, with influenza infection onset, several processes occur that might impact the antibacterial innate immune response, rendering both the upper airways and lungs susceptible to subsequent bacterial infiltration, leading to increased bacterial load and mortality (Hillyer et al., 2004; Ishikawa et al., 2016). These processes include inhibition by type I interferons (IFNs) and depletion of alveolar macrophages.

#### Influenza-induced type I IFNs might interfere with antibacterial responses

The influenza non-structural protein 1 (NS1) is produced by infected cells and can modulate innate immune pathways including IFN signaling (Hale et al., 2008; Bucasas et al., 2013). Besides playing a central role in the host antiviral response (Theofilopoulos et al., 2005), type I IFNs can also disrupt lung immune responses to bacteria (Kukavica-Ibrulj et al., 2009; Techasaensiri et al., 2010; Kimaro et al., 2013; Lee et al., 2015).

In general, all cells are equipped with specific receptors, known as pattern-recognition receptors, to detect the presence of pathogens such as viruses and bacteria. The Toll-like receptor family comprises this class of receptors and includes receptors to viral and bacterial products. Type I IFNs are produced following the recognition of influenza nucleic acids by these receptors (Tian et al., 2012), which probably functions by suppressing the normal phagocytic activity and early innate responses of macrophages and neutrophils, which would normally help to clear the bacteria from the lungs (Sun and Metzger, 2008; Shahangian et al., 2009).

Furthermore, type I IFNs can inhibit Type 17 T cells (Kudva et al., 2011; Nakamura et al., 2011), which play an important role in clearance of pulmonary pathogenic bacteria. The immunity of Type 17 T cells depends on IL-17, IL-22, and IL-23. However, type I IFNs can decrease these cytokines. Meanwhile, type I IFNs can decrease the production CCL2, which is required for macrophage recruitment (Nakamura et al., 2011). Interestingly, the antibacterial innate immune response is recovered until type I IFN levels return to baseline (Lee et al., 2015).

#### Influenza viruses deplete alveolar macrophages

Alveolar macrophages are vital for the first cellular line of defense against inhaled antigens, and account for >90% of all cells in the bronchoalveolar lavage fluid of uninfected respiratory tissue (Vermaelen and Pauwels, 2004). However, airway-resident alveolar macrophages are specifically targeted by influenza viruses during the primary stages of infection (Ghoneim et al., 2013). These depleted alveolar macrophages can be replaced over next 2 weeks by the proliferation and differentiation of macrophages of other classes. Therefore, there is a window of primary susceptibility to bacterial infection (Douek et al., 2009). For example, pneumococcal colonization density increases in mice 1 week after inoculation of influenza due to the absence of macrophages, which are necessary to clear the infection upon single-agent inoculation (Zhang et al., 2009). Moreover, influenza infection can inhibit G-CSF secretion; this decrease in G-CSF might reduce myeloperoxidase activity. Interestingly, the digestion ability of phagocytized bacteria of Neutrophils dependents on the activity of myeloperoxidase (Anderson et al., 1985; Ishikawa et al., 2016).

### Influenza viruses help to provide more binding receptors and sites for bacteria

After influenza infection is established, most viral subtypes replicate in the mucosal epithelial cells of the upper respiratory tract, providing more receptors for bacteria (Hatta et al., 2007; Nakamura et al., 2011). However, some viral subtypes can target both upper and lower respiratory tract tissues (Shinya et al., 2006; Childs et al., 2009; Maines et al., 2009; Munster et al., 2009). In particular, when viral infection precedes the presence of bacteria, access to otherwise inaccessible receptors in the lower respiratory tract might be available to invading bacteria (McCullers and Bartmess, 2003). Here, we consider three main mechanisms associated with this phenomenon.

First, influenza virus proteins can contribute. Neuraminidase, present on the envelope of the influenza virus, is responsible for sialidase activity, required by the virus for budding. After neuraminidase cleaves sialic acid from the termini of glycochains, cryptic receptors on host cells become exposed, and bacteria such as pneumococci can adhere (Foster and Hook, 1998; McCullers and Tuomanen, 2001). Furthermore, disrupted sialylated mucins can provide decoy receptors for bacteria (Plotkowski et al., 1986, 1993). For example, substantial numbers of epithelial cells can be destroyed by virulent viruses such as the mouse-adapted influenza strain PR8, resulting in exposed sites for bacteria to attach in the tracheobronchial tree (Plotkowski et al., 1986; McCullers and Rehg, 2002). Interestingly, neuraminidase proteins are not restricted to viruses. Some bacteria like *Streptococcus pneumoniae* also provide neuraminidases to access receptors and inhibit host defenses by cleaving sialic acids from protective mucins, allowing efficient infection of host lungs (Camara et al., 1991).

Second, the host inflammatory response to influenza infections can provide additional receptors. The host inflammatory response can alter not only the regulatory state, but also the surface display of multiple proteins, to facilitate pneumococcal invasion (Cundell and Tuomanen, 1994; Miller et al., 2007).

Third, adherence sites might also be provided during wound recovery in the airways (Plotkowski et al., 1993; de Bentzmann et al., 1996; Martin and Leibovich, 2005). There are some differences in terms of the wound recovery between common infection and co-infection. During co-infection with complex pathogens, reduced damage tolerance will occur; however, as a result, repair efficiency will decrease compared to that with a typical infection. This is also one of the causes of increased mortality with co-infection. Although some progress has been made, many potential mechanisms still need to be elucidated. Apical receptors including asialylated glycans (for example, GalNacβ1-Gal) or α5β1 integrins can be expressed on the surfaces of injured cells or those in an intermediate state of differentiation. Bacteria such as *Staphylococcus aureus* or *Pseudomonas aeruginosa* can adhere to receptors (Puchelle et al., 2006). Furthermore, bacteria such as *S. pneumoniae, Haemophilus influenzae*, or *S. aureus* can bind to exposed areas of incomplete healing via traditional adhesins. These exposed areas can be covered by basement membrane elements such as fibrin and fibrinogen deposition, laminin, or type I and IV collagen. This phenomenon has been observed in the clinics; patients can be easily infected by bacteria while recovering from primary illness (Louria et al., 1959; Peteranderl et al., 2017). In addition, many bacterial virulence factors can attach to elements of the extracellular matrix or basement membrane (Foster and Hook, 1998; McCullers and Tuomanen, 2001).

## Bacterial co-infections following influenza infection results in morbidity and mortality

### Influenza causes substantial lung epithelial damage

The viral cytotoxin PB1-F2, not present on all subtypes of influenza, is a non-structural protein encoded by an alternative reading frame on genomic segment 2 of influenza A. PB1-F2 is encoded by a small, variable open reading frame in *PB1* that exists in most influenza viruses. This protein is capable of activating AP-1 transcription factors via ERK1/2 kinase, and was confirmed to be a determinant of virulence. The length of PB1-F2 differs according to subtype; full-length PB1-F2 is 90 aa (amino acids). However, that of the A/Puerto Rico/8/24 strain was found to be 87 aa. Most avian influenza viruses have complete PB1-F2-encoding genes. After 1947, PB1-F2 of the H1N1 subtype was found to be cleaved at position 57, and that of the classical swine H1N1 subtype was determined to be cleaved at 11, 25, and 34 aa, which results in reduced viral pathogenicity. Since 1968, variations in the PB1-encoding gene of the H3N2 subtype gradually stabilized, and PB1-F2 truncation gradually represented a novel evolutionary feature. The 2009 H1N1 strain did not show any significant antigenic and pathogenic variation. Patient infection after obvious gastrointestinal symptoms is not associated with viral virulence, but might be caused by individual-specific properties. When viral infection is established, PB1-F2 can induce apoptosis, mediated by mitochondrial permeabilization (McAuley et al., 2007, 2010; Alymova et al., 2011; Leymarie et al., 2013), ultimately providing nutrients to invading opportunistic bacteria, following cytopathic damage and disruption of surfactant in the lungs. Consequently, inhaled or commensal bacteria can become overgrown, adversely affecting host survival (Loosli et al., 1975). In 2016, observations (Sun et al., 2016) showed that effective antibiotic treatment of clinical post-influenza bacteria depends on nicotinamide adenine dinucleotide phosphate oxidase 2 (Nox2), and that a balance exists between Nox2-dependent antibacterial immunity and inflammation. However, influenza infection might disrupt this balance and increase susceptibility to bacterial infection.

### Synergism during influenza/bacterial co-infections

Both influenza and bacteria contribute to the immunopathogenicity of co-infection. For example, expression of PB1-F2 has been associated with excessive inflammatory responses, which can lead to increased cellular infiltration of the lungs and airways, together with a cytokine storm (Conenello et al., 2007; McAuley et al., 2007, 2010). Interestingly, it was proposed that nascent hemagglutinin can be cleaved, from its primary state to a fusion-active complex, by bacterial proteases from *S. aureus* (Tashiro et al., 1987), which might increase influenza viral titers and spread.

Likewise, bacterial cytotoxins such as pneumolysin and Panton-Valentine leukocidin (PVL), can also contribute to immunopathogenicity (Rogolsky, 1979; Tuomanen et al., 1995; Loffler et al., 2013; Wolf et al., 2014). Bacterial components that lead to exacerbated cell death, associated with pore formation or enhanced inflammatory signaling, might synergize with influenza virulence factors (Boulnois et al., 1991). In addition, multiple innate immune mechanisms that involve pathogen recognition receptors also generate inflammatory responses to influenza and/or bacteria (Koppe et al., 2012; Ramos and Fernandez-Sesma, 2012). These inflammatory pathways work together, leading to synergistic activation of the immune system and increased mortality (Joyce et al., 2009; Bucasas et al., 2013; Kimaro et al., 2013).

Moreover, certain pathogenic virulence factors are only evident with the presence of a co-pathogen, as is the case for a mouse model of influenza and *H. influenzae* co-infection (Wong et al., 2013). However, it has not been possible to identify synergistic pathogenicity genes that facilitate co-infections using traditional virulence screens during single-agent infections (Bellinghausen et al., 2016).

### Influenza compromises host tolerance

The concept of tolerance used here is different from “immunological tolerance,” which is described as a state of unresponsiveness to self-antigens. Tolerance, in the current context, can reduce the negative impact of pathogen outgrowth and immunopathology, but can be compromised by the influenza virus, resulting in mortality even in the context of effective resistance.

For example, recent observations (Jamieson et al., 2013) demonstrated that influenza can compromise tolerance to tissue damage and result in mortality when mice were co-infected with *Legionella pneumophila*. It was shown that lethal synergy can be independent of impaired resistance to either influenza or *L. pneumophila*. Notably, this is different from previously discussed co-infections with influenza and opportunistic bacterial pathogens. Moreover, during the 1918 influenza outbreak, it was observed that human mortality was not directly related to infection rates (Shanks and Brundage, 2012). This study also demonstrated that lethal synergy between the influenza and *L. pneumophila* is unlikely to be due to failed immune resistance to either agent (Mendel et al., 1998).

## Discussion

The mechanisms of enhanced mortality following influenza-associated bacterial co-infections not only include failed antibacterial resistance and synergistic immunopathogenicity, but also failed tolerance. As a relatively new concept in animal immunity, tolerance has been largely overlooked and deserves further consideration regarding its effect on co-infection (Medzhitov et al., 2012).

Upon infection by a virus and/or bacterium, the host can protect itself through two distinct strategies, resistance and tolerance. Resistance is based on pathogen detection and elimination, whereas tolerance can restrict harm caused by the pathogen burden. However, virus/bacterial co-infections results in some difficulty in the treatment of either infection. For example, it is unknown whether antiviral treatments that viral load have any effect on concurrent bacterial infection (McCullers, 2004; Naguib et al., 2017). Similarly, the efficacy of treatments to counteract host inflammatory responses to bacterial co-infection is unknown (Kudva et al., 2011; Arduin et al., 2017). Therefore, distinguishing between failed resistance and failed tolerance might be of vital importance for the selection of therapeutic approaches to treat the primary problem.

Considering that mortality during co-infection can be decreased when bacterial infection occurs before influenza challenge, albeit through unknown mechanisms (McCullers and Rehg, 2002; Wang et al., 2013), it is possible to develop novel recombinant vaccines that include both influenza and bacterial antigens. Ideally, this vaccine would elicit cross-reactive antibody responses to both influenza and bacteria. Furthermore, such vaccines would represent an appealing alternative to classical inactivated vaccines.

Therefore, understanding the mechanisms involved in the synergy between viral and bacterial co-infection will facilitate the design of novel therapeutic approaches for the prevention of elevated mortality associated with bacterial co-infections following influenza infection (Boianelli et al., 2016; McDonald et al., 2017; Smith, 2017).

## Author contributions

All author participated in developing the hypothesis and collaborated in writing and reviewing of the article.

### Conflict of interest statement

The authors declare that the research was conducted in the absence of any commercial or financial relationships that could be construed as a potential conflict of interest.
